# Bias Calibration of Optically Pumped Magnetometers Based on Variable Sensitivity

**DOI:** 10.3390/s25020433

**Published:** 2025-01-13

**Authors:** Jieya Chen, Chaofeng Ye, Xingshen Hou, Yaqiong Niu, Limin Sun

**Affiliations:** 1 Shanghai Institute of Microsystem and Information Technology, Chinese Academy of Sciences, Shanghai 200050, China; chenjy2022@shanghaitech.edu.cn; 2 School of Information Science and Technology, ShanghaiTech University, Shanghai 201210, China; houxsh2023@shanghaitech.edu.cn (X.H.); niuyq@shanghaitech.edu.cn (Y.N.)

**Keywords:** OPM, bias calibration, biomagnetic field measurement, magnetic field compensation

## Abstract

Optically pumped magnetometers (OPMs) functioning in the spin-exchange relaxation-free (SERF) regime have emerged as attractive options for measuring weak magnetic fields, owing to their portability and remarkable sensitivity. The operation of SERF-OPM critically relies on the ambient magnetic field; thus, a magnetic field compensation device is commonly employed to mitigate the ambient magnetic field to near zero. Nonetheless, the bias of the OPM may influence the compensation impact, a subject that remains unexamined. This paper introduced an innovative bias calibration technique for OPMs. The sensitivity of the OPM was altered by adjusting the cell temperature. The output of the OPM was then documented with varying sensitivity. It is assumed that the signal exhibits a linear correlation with the environmental magnetic field, and the statistical characteristics of the magnetic field are identical for both measurements, upon which the bias of the OPM is assessed. The bias was subsequently considered in the feedback magnetic field compensation mechanism. The results indicate that this technique might successfully reduce environmental magnetic fluctuations and enhance the sensitivity of the OPM. This technique measured the magnetic field produced by the human heart, confirming the viability of the ultra-weak biomagnetic field measurement approach.

## 1. Introduction

Biological magnetic fields denote the magnetic fields produced by biological processes, generally arising from electrical activities within the body, including heartbeats, neuronal activity in the brain, and muscular contractions. Assessing these magnetic fields provides a non-invasive technique for tracking bio-electrical activity, with significant applications in medicine and neurology [1,2,3,4,5]. Magnetocardiography (MCG) and magnetoencephalography (MEG), for example, are widely used to diagnose and study heart diseases and brain disorders, respectively [6,7,8,9,10]. Detecting these signals presents a significant technical challenge due to the extremely weak magnetic fields produced by living organisms, typically on the order of picotesla (pT) or even femtotesla (fT).

One of the main instruments for measuring the weak biological magnetic fields is superconducting quantum interference devices (SQUIDs), which require operation in a low temperature superconducting environment. This limits their widespread use in clinical and portable applications [11]. Researchers have studied alternative sensors, such as optically pumped atomic magnetometers (OPMs), to achieve high-sensitivity measurements of weak magnetic fields at room temperature. OPMs utilize the response of alkali metal atomic gases to laser light to measure magnetic fields [12]. This characteristic makes OPM an ideal tool for measuring weak biological magnetic fields. The sensitivity of OPMs has approached that of SQUIDs [13,14,15], making them suitable for detecting weak electromagnetic signals generated by biological processes. Compared with SQUIDs, OPMs are cheaper and easier to implement in portable applications [16].

OPMs exhibit super sensitivity when operating in the spin-exchange relaxation-free (SERF) regime. In this regime, spin-polarization relaxation caused by spin-exchange collisions is suppressed, significantly enhancing the sensitivity of the OPM [17,18]. However, the performance of a SERF-OPM is highly contingent upon the surrounding magnetic field environment, with zero magnetic field conditions being essential for optimizing its sensitivity [19]. External magnetic fields and environmental noise can obscure or even overwhelm weak magnetic signals. Therefore, it is imperative to establish an appropriate magnetic field environment.

To achieve a near-zero magnetic field environment, passive magnetic shielding is commonly employed to reduce the magnetic field to the nanotesla (nT) level. This is followed by active magnetic field compensation to further nullify the remaining magnetic field [20,21,22,23,24]. Wang et al. presented a method for single-beam OPMs to acquire three-axis magnetic field information by applying modulation magnetic fields at varying frequencies along two sensitive axes [25]. Li et al. developed a method for three-axis magnetic field measurement employing orthogonal transverse modulation fields, which provided high sensitivity and a broad bandwidth [26]. Yan et al. created a high-sensitivity three-axis closed-loop OPM with a sensitivity of 30 fT/Hz^1/2^ [27]. Long et al. mitigated the cross-axis coupling effect in a dual-beam unmodulated SERF-OPM by employing proportional-integral (PI) closed-loop control to compensate for the background magnetic field [28]. Wang et al. introduced a technique to concurrently correct for gradient fields and residual magnetic fields, enhancing the measurement sensitivity of the dual-beam OPM [29]. Fang et al. tuned the probing beam wavelength and accounted for the pumping effect to enable three-axis magnetic field correction, enhancing the sensitivity of the OPM to roughly 4 fT/Hz^1/2^ [30]. Moreover, utilizing a three-axis coil array for magnetic field calibration successfully eradicated cross-axis crosstalk among sensors, enhancing the uniformity of the OPM array and refining spatial localization precision [31]. The dual-layer planar coil design efficiently mitigated spatially variable magnetic field interference via dynamic magnetic field nulling technology, allowing the OPM to conduct high-quality MEG recordings in an unconstrained environment [32,33].

Magnetic field compensation algorithms often depend on identifying the sensor signal’s zero point to modify the magnetic field. However, a circuit that processes and amplifies the output of an OPM inevitably introduces bias. The zero output of an OPM does not accurately reflect a zero magnetic field. Notwithstanding a comprehensive examination of the literature, the influence of OPM bias has not been thoroughly explored. This work introduced a bias calibration technique for OPMs utilizing variable sensitivity, hence, improving the accuracy and stability of magnetic field adjustment.

## 2. Principle

### 2.1. Operating Principle of the SERF-OPM

**Figure 1:**
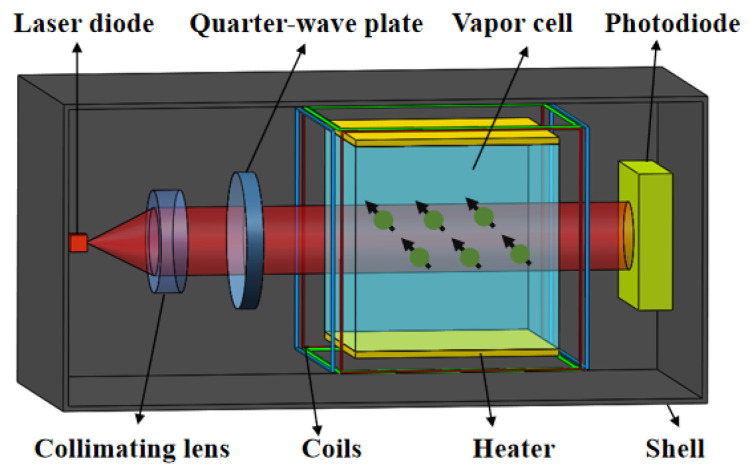
Schematic diagram of the of the OPM sensor.

The SERF-OPM utilized in this study was developed by our laboratory. The dimensions of the OPM are 20 × 20 × 40 mm^3^. The structure diagram of the sensor is shown in Figure 1. It employs a vertical cavity surface emitting laser (VCSEL) diode as the light source. The light beam is collimated into a cylindrical shape with a diameter of approximately 2 mm, then passes through a λ/4 waveplate. The light passes through a vapor cell containing enriched ^87^Rb atoms, helium, and nitrogen gases. The interaction between the light and atoms induces spin precession. The evolution of the atomic spins and their response to external magnetic fields are described by the Bloch equation [34]:

(1) $$\frac{d\vec{P}}{dt}=\frac{1}{q}\left[\gamma_{e}\vec{B}\times \vec{S}+R_{op}\left(s\hat{z}-P\right)-R_{rel}\vec{P}\right]\;$$

where $\vec{P}=\left(P_{x},P_{y},P_{z}\right)$ is the electron polarization of the atoms, *q* is the nuclear slowing-down factor, $\gamma_{e}$ is the gyromagnetic ratio, $\vec{B}=\left(B_{x},B_{y},B_{z}\right)$ is the magnetic field vector, *s* is the average photon polarization of the pump beam, $R_{op}$ is the laser pump rate, and $R_{rel}$ is the spin-relaxation rate.

With the existence of magnetic fields, the steady state of polarization along the pumping axis $P_{z}$ is written as

(2) $$P_{z}=P_{0}\frac{{\left(\Gamma /\gamma_{e}\right)}^{2}+{\left(B_{z}\right)}^{2}}{{\left(\Gamma /\gamma_{e}\right)}^{2}+{\left(B_{x}\right)}^{2}+{\left(B_{y}\right)}^{2}+{\left(B_{z}\right)}^{2}}\;$$

where $\Gamma =R_{op}+R_{rel}$ is the total relaxation rate and $P_{0}=R_{op}/\Gamma$ is the equilibrium polarization.

To suppress the effect of 1/f noise, a modulation magnetic field $B_{xm}\cos\left(\omega_{xm}t\right)$ is applied along the x-axis. Let $B_{xs}$ denote the magnetic field to be measured along the x-axis and *V* is the output voltage signal. The output is written as [35]:

(3) $$V\propto \frac{\gamma_{e}R_{op}J_{0}\left(\mu \right)J_{1}\left(\mu \right)}{{\left(R_{op}+R_{rel}\right)}^{2}+{\left(\gamma_{e}B_{x}\right)}^{2}}B_{x}\;$$

where $J_{0}$ and $J_{1}$ are the Bessel functions of the first kind with the modulation index $\mu =\gamma_{e}B_{xm}/\left(q\omega_{xm}\right)$, $B_{x}=B_{x0}+B_{xs}$. With $\gamma_{e}B_{x}\ll \left(R_{op}+R_{rel}\right)$, the output of the OPM is approximately linearly correlated with the magnetic field $B_{x}$.

A zero-field condition is necessary for a SERF-OPM. The OPM system’s circuit may introduce a certain level of bias. To obtain in situ magnetic field adjustment for OPMs, most previous systems used an iterative optimization method to cancel out the OPM output without taking bias into account. The bias induces a field shift in the SERF-OPM, and the OPM does not operate in an authentic zero-field environment. To address this issue, this paper proposed a method for assessing the bias of an OPM. To simplify the analysis, the output voltage of the OPM is written as a linear function of the magnetic field.

(4) $$v_{x}=k\left(B_{x}+B_{0}\right)+C$$

where $v_{x}$ is the output voltage, *k* is the sensitivity coefficient of the OPM, $B_{x}$ is the magnetic field to be measured along the x-axis, $B_{0}$ is the environment magnetic field in the magnetic shielding room (MSR), and *C* is the bias.

### 2.2. Calculation of the Bias

The temperature of the atom cell ($T_{c}$) affects the sensitivity of the OPM by influencing the density of the alkali metal atoms and related spin relaxation mechanisms [36]. In other words, *k* is different for different cell temperatures. Using this principle, we could alter the sensitivity of the OPM by adjusting the cell temperature. In the experiment, the OPM operated at two different atom cell temperatures, namely $T_{c}=T_{1}$ and $T_{c}=T_{2}$. Assume the sensitivity coefficients are $k_{1}$, $k_{2}$ for two different atom cell temperatures, respectively. Then, a magnetic field with varying strength was applied to the OPM and the output was recorded. The values of $k_{i}\left(i=1,2\right)$ were calibrated using a linear fit. The environmental magnet field was then measured without any applied magnetic field, namely $\left(B_{x}=0\right)$, in which case the formula was written as (5).

(5) $$v_{x}=k_{i}B_{0}+C\;i=1,2\;$$

The environmental magnetic field $B_{0}$ is written as (6).

(6) $$B_{0}=\frac{v_{x}-C}{k_{i}}\;i=1,2\;$$

It is particularly noteworthy that the sources of the bias *C* are independent of the alkali-metal vapor density or spin relaxation processes. Therefore, its statistical characteristic does not depend on the cell temperature. The statistical characteristic of *C* remains stable and predictable. The experiments were conducted in an MSR, which was constructed with materials with high magnetic permeability, significantly attenuating the Earth’s magnetic field [37,38]. The MSR suppressed the environmental magnetic field down to a level of a few nanoteslas. The magnetic field inside the MSR is typically caused by changes in external magnetic fields or residual magnetization of materials within the room. It is reasonable to assume the field fluctuations have a similar statistical distribution, thus

(7) $${\bar{B}}_{0}{|}_{T_{c}=T_{1}}={\bar{B}}_{0}{|}_{T_{c}=T_{2}}$$

Substituting (6) into (7), the bias *C* was derived.

(8) $$C=\frac{k_{2}{\bar{v}}_{x1}-k_{1}{\bar{v}}_{x2}}{k_{2}-k_{1}}$$

### 2.3. Magnetic Field Compensation with Bias

Upon determining the value of *C*, a proportional–integral–derivative (PID) controller was employed to compensate for the magnetic field fluctuation by applying the corresponding current to the coil to cancel the magnetic field. The output voltage of the OPM was driven towards the target value corresponding to a zero magnetic field. The PID controller compared the desired target value (*C*) with the actual value of the system. The difference between these two values was called the error value, denoted as e(t).

(9) $$e\left(t\right)=v_{x}\left(t\right)-C\;$$

The PID controller worked as follows.

(10) $$i\left(t\right)=K_{p}e\left(t\right)+K_{i}\int_{0}^{t}e\left(t\right)\;d\tau +K_{d}\frac{de(t)}{dt}\;$$

where $K_{p}$, $K_{i}$, and $K_{d}$ are the proportional, integral, and derivative gain coefficients of the PID controller, respectively. i(t) is the compensation current. The PID controller iteratively adjusts the compensating magnetic field until the sensor output satisfies $v_{x}=C$, indicating that the sensor is in a zero magnetic field environment.

## 3. Experiment Setup

### 3.1. System Diagram

**Figure 2:**
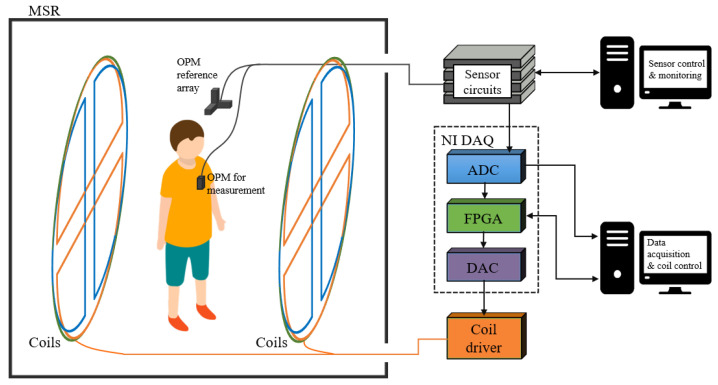
Diagram of the experimental setup.

An experiment system has been constructed to test the feasibility of the method. The diagram of the experiment system is shown in Figure 2. The system was constructed inside an MSR composed of three layers of permalloy and one layer of aluminum, with internal dimensions of 2.1 × 2.1 × 2.1 m^3^. Three OPMs were used in the experiment to monitor the environmental magnetic field, which were placed in the uniform region of the coils to measure the three components of the magnetic field: $B_{x}$, $B_{y}$, and $B_{z}$.

### 3.2. Compensation Coils

**Figure 3:**
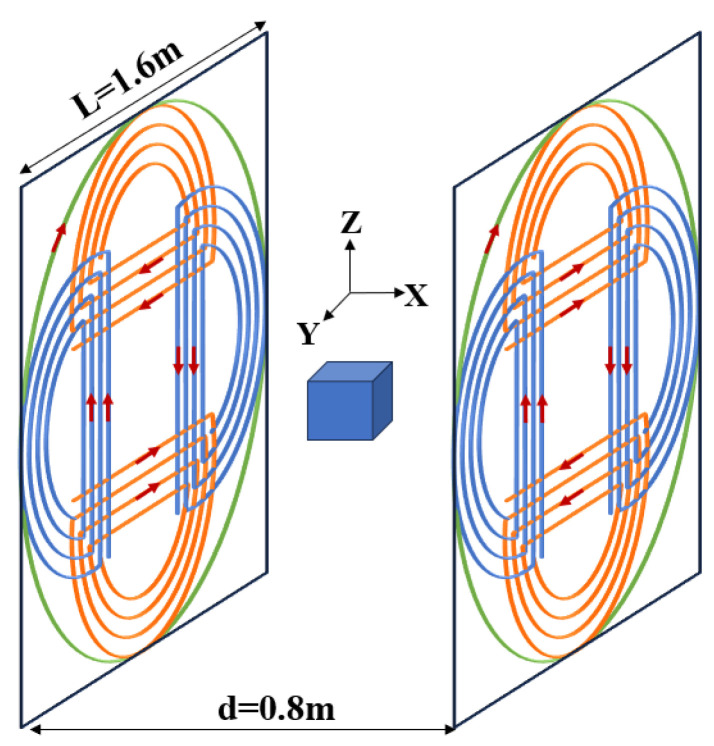
Illustration of the magnetic field compensating coils. Distinct colors represent the coils along different axes: green for the x-axis, blue for the y-axis, and orange for the z-axis. The arrows denote the directions of the currents.

The diagram of the magnetic field compensation coil is shown in Figure 3. The coils were installed on either side of the measurement area, forming a dual-plane system.

The coil system had two types of coils: the Helmholtz coil and double D-shaped coils. The Helmholtz coil was employed to mitigate the magnetic field along the x-axis, whereas the double-D-shaped coils were utilized to counteract the magnetic fields along the y- and z-axes.

**Figure 4:**
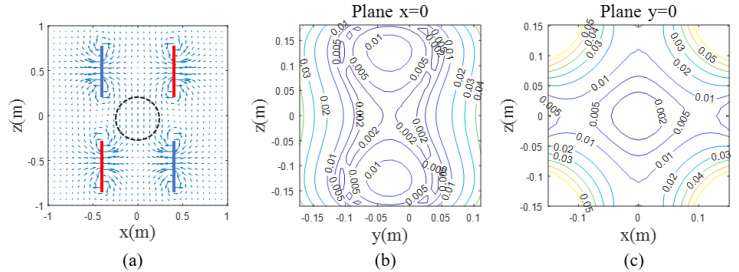
Magnetic field distribution and uniformity of the double D-shaped Coils. (**a**) The position of the double D-shaped coils and the magnitude and direction of the generated magnetic field. Blue and red lines represent the positions where the coils were placed. (**b**,**c**) The uniform region of the magnetic field in the x = 0 plane and y = 0 plane. The colors of the lines indicate the degrees of uniformity.

The double D coil structure, as shown in Figure 3 with the orange coil, consisted of two semicircular rings, one on top and one on the bottom. The direction of current flow is indicated by the red arrows in the diagram. The magnetic field generated in the target area is shown in Figure 4a. The region enclosed by the black circle at the center generates a uniform magnetic field. The thick lines represented the positions of the D-shaped current loops, and each color corresponds to a different direction of current. The y-axis uniform field coil was similar to this and could be obtained by rotating the z-axis uniform field coil by 90°.

The coils were affixed to two acrylic plates, each measuring 1.6 m by 1.6 m. Each plate was supported by an aluminum alloy framework and positioned in the center of the MSR. The coil patterns were produced on paper, affixed to the acrylic plates, and subsequently twisted with an enameled copper wire measuring 0.56 mm in diameter.

To analyze the uniformity of the magnetic field generated by the coils, the magnetic field uniformity ε is defined as follows:

(11) $$\varepsilon \left(x,y,z\right)=\frac{\left|B(x,y,z)-B(0,0,0)\right|}{B(0,0,0)}$$

where B(0,0,0) is the magnetic field strength at the center point, and B(x,y,z) is the magnetic field strength at any observation point.

**Figure 5:**
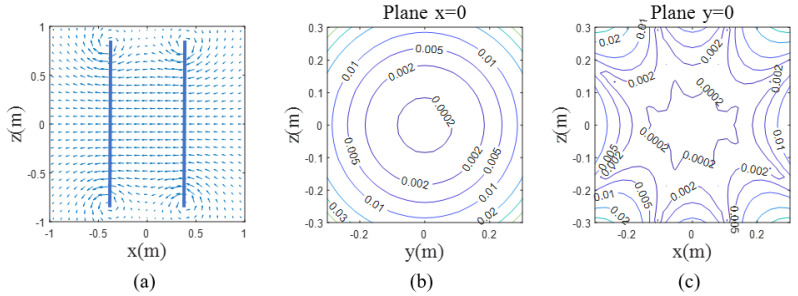
Magnetic field distribution and uniformity of the Helmholtz coils. (**a**) The position of the Helmholtz coils and the magnitude and direction of the generated magnetic field. (**b**,**c**) The uniform region of the magnetic field in the x = 0 plane and y = 0 plane. The colors of the lines indicate the degrees of uniformity.

Based on the parameters of the double D-shaped coil, the magnetic field uniformity distribution and its contour lines were calculated, as shown in Figure 4. The magnetic field uniformity in the x=0 plane exhibited a peanut shape, with the center of symmetry of the magnetic field shifted 3 cm in the negative y-direction. This was due to the slight asymmetry of the helical structure. Therefore, when connecting the coils, they were shifted 3 cm in the positive y-direction to ensure that the uniform magnetic field region was centered. There was a 24×24 cm^2^ area where the magnetic field uniformity was within 5%. Similarly, the magnetic field distribution and uniformity contours of the Helmholtz coil are shown in Figure 5, revealing a larger uniform region that ensured the magnetic field uniformity within a 60×60 cm^2^ area remained within 5%.

Consequently, when three pairs of coils were configured in a dual-plane arrangement, a homogeneous magnetic field could be produced in the middle region with a 24×24×24 cm^3^ volume. Attaining a homogeneous magnetic field is essential for precise magnetic measurements, particularly in sensitive biomagnetic detection. Through the optimization of coil specifications and construction, an approximately uniform magnetic field distribution was achieved inside the measuring space.

### 3.3. Control and Data Acquisition

The outputs of the OPMs were recorded by a data acquisition system (NI-7856R from National Instruments Corporation). The sample rate was 1 kHz. To eliminate the residual magnetic field inside the MSR, three controllers were used to calculate the optimal current through each plane coil winding, and the voltage outputs to drive the coils were provided by the NI-7856R DAC. Each pair of coils in the dual-plane coil system was connected in series to a 10 V coil driver. An appropriately sized resistor was placed in series with each coil circuit, and the maximum output voltage of the coil driver (10 V) generated a magnetic field of approximately 50 nT.

## 4. Experimental Results and Discussion

### 4.1. Parameters Estimation

**Figure 6:**
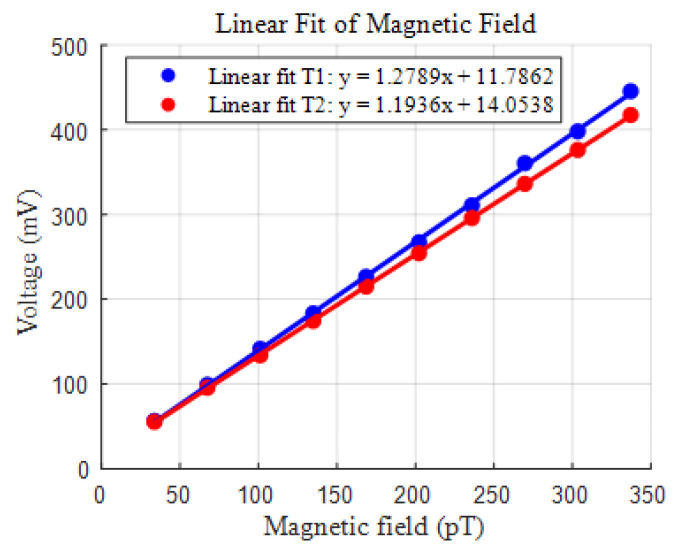
Fitted signal vs. magnetic field curves at different temperatures.

The OPM functions effectively within a temperature range of 125 °C to 155 °C. The sensor’s sensitivity is affected by the temperature of the cell, with 138 °C tested as the optimal operating temperature. Therefore, we selected 138 °C as one of the experimental temperatures. Furthermore, it is observed that altering the cell temperature by 2 °C yields a measurable change in sensitivity. Thus, the two temperatures of 138 °C and 140 °C were employed in the experiment. The sensitivities of the sensor were measured at different temperatures. The results are shown in Figure 6.

**Figure 7:**
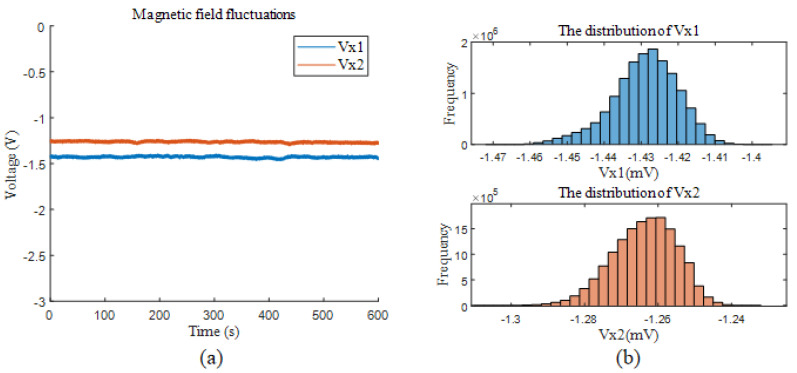
Magnetic field fluctuations and their distribution at different temperatures. (**a**) Residual magnetic field fluctuations at two temperatures. (**b**) Histogram of $v_{x1}$ and $v_{x2}$.

The calculated sensor sensitivity coefficients were 1.2789 nT/V and 1.1936 nT/V, respectively. Next, the OPM output was recorded for 10 min to measure the environmental magnetic field. The time-domain plots of the results are shown in Figure 7a. The distributions of the signals are represented in Figure 7b as histograms. The histograms illustrate the occurrence frequency of the signals within the corresponding voltage intervals, reflecting their statistical characteristics. Observing the distributions revealed almost consistent fluctuations, indicating that the signals maintain relatively stable patterns.

The bias of the OPM with its sensitive axis oriented along the x-axis was calculated as C=1.0464 V. After determining the bias of the OPM *C*, a PID controller utilized the bias as the setting value to compensate for the magnetic field.

**Figure 8:**
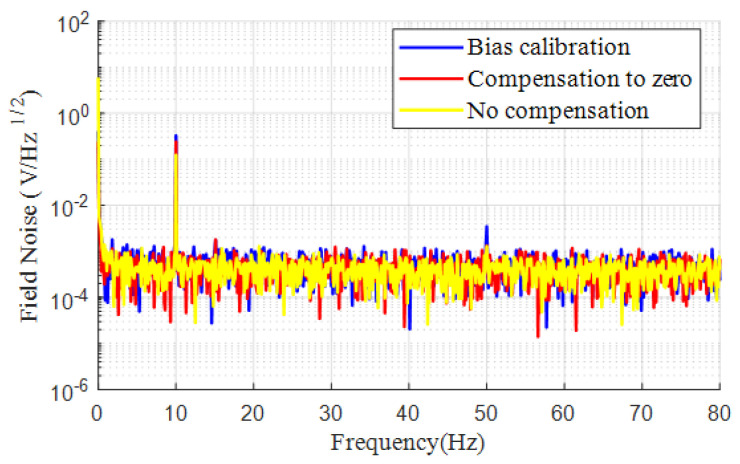
Comparison of the magnetic field measurement sensitivities without magnetic field compensation, with the DC residual magnetic field compensation to zero, and with bias calibration methods.

The sensitivity of the OPM post-compensation, accounting for bias, was compared with the method of simply calibrating the sensor’s output to zero. The temperature of the cell was consistently maintained at 138 °C. Figure 8 presents the results. The sensitivities of the OPM under the 10 Hz 100 pT calibration magnetic field were 123.99 mV/100 pT, 241.26 mV/100 pT, and 327.21 mV/100 pT for the cases without compensation, compensating the sensor’s output to zero, and adjusting the sensor’s output to the bias, respectively. It is evident that the implementation of the proposed method enhanced the sensitivity of the OPM by almost 2.5 times relative to the conventional technique of nullifying the magnetic field.

### 4.2. Magnetic Field Fluctuation Suppression Experiment

**Figure 9:**
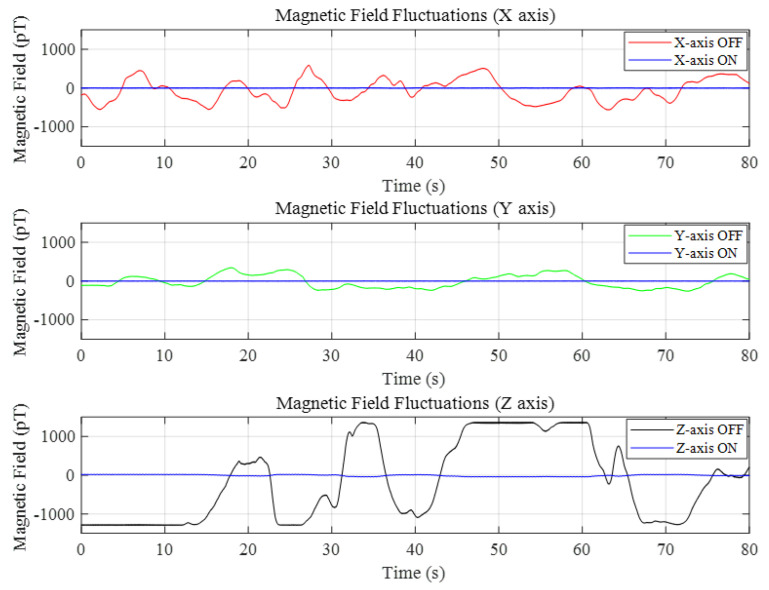
Compensation effects in three directions.

To evaluate the performance of the OPM and illustrate the efficacy of the PID adjustment in mitigating magnetic field fluctuations, the sensor was positioned within an unoccupied MSR, and its output was documented. The experiment was performed in an MSR situated in a building’s basement, adjacent to a subway line. The subway generated a significant magnetic field fluctuation with each passage. Data were recorded over 80 s with the magnetic field compensation system in both “on” and “off” states. The results are presented in Figure 9. The results demonstrated that without the background magnetic field suppression system, the field fluctuations along the x-axis (north–south) and y-axis (east–west) were 1.16 nT and 610 pT, respectively, while the fluctuations along the z-axis (vertical) were excessively large, resulting in the saturation of the OPM output. Upon activating the dynamic switch for the background magnetic field, the field fluctuations were reduced to 29.63 pT, 17.23 pT, and 71.56 pT along the x-, y-, and z-axes, respectively.

### 4.3. Magnetocardiography Measurement

To verify the feasibility of the technique for ultra-weak biomagnetic field measurement, the magnetic field generated by a human heart was measured. The experiment setup is shown in Figure 2. The OPM was mounted on a stand, and the subject leaned forward toward the sensor for the measurements. During the recording, the subway line was active, resulting in considerable magnetic field disturbance. The recorded signal was processed using a fourth-order Butterworth bandpass filter with a frequency range of 0.5–15 Hz.

**Figure 10:**
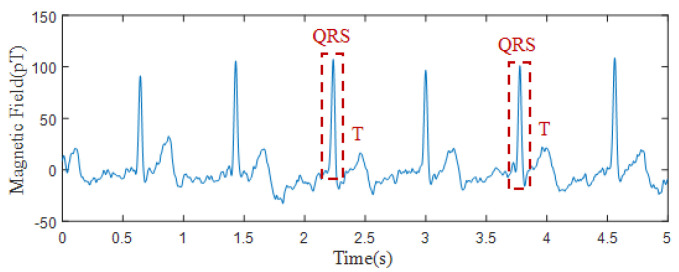
Magnetic field signals of the heart as measured by OPM.

Figure 10 illustrates the recorded magnetic field signal of the heart acquired by the experimental apparatus. The collected signals exhibited distinct periodic oscillations that aligned with the heartbeat, occurring approximately every 0.8 s, indicative of standard cardiac activity. The QRS complex was distinctly recognizable, and in the majority of trials, the T wave was also prominently discernible. The detected signal aligned with previous MCG research [6], indicating that the technology can identify the magnetic fields generated by the heart despite the interference from the subway.

### 4.4. Discussion

This approach is theoretically applicable to all OPMs whose sensitivity relies on the cell temperature. Future studies could explore the applicability of this approach to a broader range of OPMs to further validate its universality. The bias estimation based on variable sensitivity depends on the regulation of cell temperature. Most commercial OPMs appear to be equipped with closed temperature control systems that do not provide user-accessible adjustment interfaces, hence, restricting the usefulness of this technology to existing devices. In conclusion, the experimental results verify the method’s viability. It is an excellent method to manage the impact of OPM bias, provided that the sensor’s sensitivity is adjustable.

This research examines the impact of a single-axis OPM’s bias on magnetic field correction. Multichannel or three-dimensional optically pumped magnetometer (OPM) array devices are crucial for biomagnetic field mapping applications.Their capacity to attain elevated spatial resolution and three-dimensional vector magnetic field measurements facilitates thorough acquisition of intricate biomagnetic signals, markedly enhancing imaging precision and source localization accuracy. OPM technology is anticipated to progress toward multi-axis measurement, sensor downsizing, and high-density multichannel setups. These advancements will significantly propel progress in biomagnetic imaging and source localization. Consequently, the relevance of this technique to more intricate OPM systems warrants additional investigation.

## 5. Conclusions

This paper presented an innovative approach for the evaluation of the bias in OPMs, substantially mitigating the influence of unknown bias signals on magnetic field compensation. This new method was implemented by adjusting the temperature of the cell to change the sensitivity of the OPM. Furthermore, the output of the OPM was documented with two sensitivities to accurately estimate the K value for precise bias calculation. It is assumed that the signal is linear to the environmental magnetic field, whose statistical properties are consistent at different temperatures. Based on this, the bias of the OPM was evaluated, which is essential for compensating the feedback of the measured magnetic field. The experiments showed that this method improved the sensitivity by more than 2.5 times compared with traditional compensation techniques. Moreover, it demonstrated strong robustness in suppressing fluctuations of the environmental magnetic field. However, it is worth noting that this method still faces certain limitations in complex environments. Future work will focus on an optimization and applications with a multi-channel array consisting of OPMs.

## Data Availability

The data presented in this study are available upon request from the corresponding author.
